# Redefining shelter: humanitarian sheltering

**DOI:** 10.1111/disa.12555

**Published:** 2023-01-26

**Authors:** Jennifer Ward George, Peter Guthrie, John J. Orr

**Affiliations:** ^1^ Research Student, Department of Engineering University of Cambridge United Kingdom; ^2^ Director of Research in Sustainable Development, Department of Engineering University of Cambridge United Kingdom; ^3^ Professor of Structural Engineering, Department of Engineering University of Cambridge United Kingdom

**Keywords:** adequate shelter, definition of shelter, displacement, emergency shelter, enabled process, humanitarian aid, internally displaced persons, non‐governmental organisation, post conflict, post disaster, refugee, self‐recovery, settlements, shelter, sheltering, temporary housing, transitional settlement, transitional shelter

## Abstract

Shelter is one of the most ‘intractable problems’ in humanitarian aid and yet there is little clarity on an overarching definition. Terminology for shelter and housing is often conflated, and the most prominent definition does not fully reflect recent progress in the Shelter and Settlements Sector. This paper explores the varying terminology utilised in definitions of shelter within humanitarian aid since 1990, reflecting on the concepts of ‘shelter’ and ‘housing’, alongside surrounding perceptions of ‘house’ versus ‘home’, and related measures of adequacy. The current, most prolific definition is also deconstructed, demonstrating ambiguity in some of terminology such as ‘dignity’ and ‘privacy’, and revealing that interpretation of this definition depends on the reader's knowledge. Lastly, a new definition of ‘sheltering’ is proposed, encompassing five key reflections: the concept of process over object; the inclusion of communities and individuals; the commonality of long‐term sheltering; the wider effects of shelter; and the impacts on host communities and environment.

## المستخلص

يوصف المأوى بأنه أحد أكثر “المشاكل المستعصية” في الإغاثة الإنسانية ومع ذلك لا يوجد إلا القليل من الإيضاح حول التعريف الشامل للمأوى. وعادة ما يكثر الخلط بين مصطلحيْ المأوى والمسكن، وعلاوة على ذلك، فإن المصطلح الأكثر استخداماً لا يعبر كلياً عن التطور المحرز مؤخراً في قطاعيْ المأوى والمستوطنات. وتشمل هذه التطورات حراكاً للتحول من “منتج” إلى “عملية”، بالإضافة إلى إدراك واعتراف بالتأثيرات الأوسع للمأوى بما في ذلك سبل المعيشة والصحة والنشاط الاقتصادي والتعليم وتقليل قابلية التأثر. تكشف هذه الورقة الستار عن تباينٍ في المصطلحات المستخدمة في تعريف المأوى في الإغاثة الإنسانية منذ عام 1990، والذي بدوره يعبر عن مفهوم “المأوى” و”المسكن” إلى جانب التصورات المحيطة بموضوع “المنزل” في مقابلة ”البيت”، وإجراءات الكفاية المتعلقة بذلك. إن التعريف الحالي الأكثر شيوعاً هو أيضاً تعريف مفكك، مما يُظهر الغموض واللبس في بعض المصطلحات من أمثال “الكرامة” و”الخصوصية”، وأيضاً يبين أن تفسير هذا التعريف يستند إلى معرفة القارئ. أخيراً، تم عرض تعريف جديد لمصطلح “الإيواء”، بما يضم خمسة اعتبارات أساسية: مبدأ الإجراءات أهم من المادة؛ وشمول المجتمعات المحلية بالإضافة إلى الفرد؛ القواسم المشتركة للإيواء على المدى الطويل؛ التأثيرات الأوسع للإيواء؛ تأثير المجتمعات المضيفة وبيئاتها. تم اقتراح هذا التعريف تزامناً مع الاحتياجات المذكورة في تعريف برنامج الأمم المتحدة للمستوطنات البشرية لما أسمته “المأوى الملائم” مع التنبيه على أن ملائمة هذه الاحتياجات والاستجابة للمأوى يجب أن يتم تحديدها بناءً على السكان المتأثرين.

الكلمات المفتاحية المأوى؛ تعريف المأوى؛ الإيواء؛ الإسكان المؤقت؛ الإيواء الانتقالي/ المرحلي؛ الإيواء في حالات الطوارئ؛ المأوى الملائم؛ المستوطنات؛ التعافي الذاتي؛ المستوطنات الانتقالية؛ بيئة المعيشة؛ النزوح؛ الإغاثة الإنسانية؛ ما بعد الكارثة؛ ما بعد النزاع؛ التعافي؛ لاجئ؛ نازح داخلي؛ منظمة غير حكومية؛ عملية تمكينية، المأوى عملية وليس منتجاً؛ المجتمعات المضيفة

## 剑桥大学

通常认为，避难所是人道主义援助中“最棘手”的问题之一，但人们对避难所的总体定义却不甚明确。避难所和住房这两个术语经常被混为一谈，此外，最广为使用的定义却没有充分反映在避难所和定居点问题上新取得的进展。这些进展包括从“产品”到“过程”的转变，以及对避难所更广泛影响的认识，包括生计、健康、经济促进、教育以及减少脆弱性。本文探讨了自1990年以来在人道主义援助中对于避难所这一概念使用的不同术语，反思了 "避难所 "和 "住房 "的概念，以及对与之相关的"房子 "与 "家 "的看法和相关的充分措施。文章解构了目前最主流的定义，阐释了一些包括“尊严”和“隐私”在内的术语的模糊性，并提出对这一定义的诠释取决于读者自身的认识。最后，文章提出了 "庇护 "的新定义，包括五个关键的思考：过程大于对象的概念；对社区和个人的包容性；长期庇护的共性；避难所的广泛影响；对收容社区和环境的影响。这一定义是结合联合国人居署对 "充分避难所 "所定义的需求提出的，但要注意的是，这些需求的适当性及对庇护的反馈应该由那些受影响的人群决定。

关键词 避难所；避难所的定义；庇护；临时住房；过渡性避难所；紧急避难所；适当的避难所；定居点；自我恢复；过渡性定居点；生活环境；流离失所；人道主义援助；灾后；冲突后；恢复；难民；国内流离失所者；非政府组织；启用过程；过程与产品；收容社区

## Introduction

In 2011, the *Humanitarian Emergency Response Review* stated that ‘providing adequate shelter is one of the most intractable problems in international humanitarian response’ (DFID, 2011, p. 25). However, despite the oft‐cited importance of shelter in post‐disaster and crisis situations (Habitat for Humanity, 2012; Felix, Branco, and Feio, 2013; Bashawri, Garrity, and Moodley, 2014; Dabaieh and Alwall, 2018), there is relatively little clarity on an overarching definition of ‘shelter’ and its associated terms in the humanitarian aid sector. Both within development and humanitarian aid, the terms for ‘shelter’ and ‘housing’ are often conflated, and alongside this, an additional term, ‘sheltering’, is used interchangeably. In addition, the ‘Shelter Sector’ has widened over time to include the term ‘settlements’, and now is referred to as the ‘Shelter and Settlements Sector’. All of these terms (housing, shelter, sheltering, and settlements) have multiple possible definitions across the development and humanitarian aid literature. These changing definitions and additions in terminology are reflective of the movement and progression of the sector, and can be explained by several drivers. First, there has been a longstanding movement in the Shelter and Settlements Sector towards recognition of shelter as a process rather than a product. This began with Ian Davis in 1978, who stated that ‘a specific product may form a *part* of the process’ but that ‘it is important to emphasise at the outset that shelter must be considered as a process, not as an object’ (Davis, 1978, p. 33). This view reflects earlier research by John Turner (1973) who referred to ‘housing as a verb’ and was later mirrored by Nabeel Hamdi (1995) who discussed the paradigm between ‘provision’ and ‘support’, particularly in development contexts. Within the Shelter and Settlements Sector, the process over object argument has since been recognised as useful across multiple sources (Kennedy et al., 2008; Davis and Alexander, 2016; IFRC, 2019). It has also resulted in proposals for process‐oriented methods of sheltering, such as cash grants and self‐recovery (Maynard, Parker, and Twigg, 2017).

Likewise, more recently, there has been a positive movement towards recognition of the wider impacts of shelter in relation to health, livelihoods, economic stimulation, education, food and nutrition, and reducing vulnerability (Kelling, 2020). In addition, an increasing body of research has focused on context‐specific shelter response, such as differing needs in urban environments or conflict situations and the importance of long‐term planning (Opdyke, Goldwyn, and Javernick‐Will, 2021). Lastly, there has been an examination of the effect of shelter on the wider critical infrastructure and host community (Fekete et al., 2021). This growing wealth of knowledge has created increasing numbers of reference terms for shelter (Brogden and Kennedy, 2021), while also demonstrating some gaps in humanitarian standards (Fekete et al., 2021).

As a result of these progressions, Sector terminology is lacking clarity. Specifically, how we should define ‘shelter’ and its purpose, and how the definition can encompass rather than exclude these aforementioned concepts. As such, this paper seeks to lay out the key definitions of shelter within humanitarian aid since 1990, deconstruct the prolific definition of a ‘habitable covered living space’, and propose a revised definition reflective of the current Shelter and Settlements Sector.

The purpose of proposing this definition is to provide a common basis upon which discourse can be conducted. The use of differing terminology across the Sector can be confusing to other clusters within humanitarian aid, act as a barrier to further progression, and hinder understanding of the broader purposes of shelter both within and without the Sector. This is of particular importance for policymakers, donors, and other stakeholders influencing shelter decisions. The lack of a singular definition of shelter incorporating progressions in the Sector means that those stakeholders must instead be required to have that knowledge already or read significantly beyond the current definition.

The paper holds no pretence of being exclusive, but rather intends to develop a useful discussion around the confusion associated with terminology and to clarify Sector developments, to act as a common basis for the diverse range of stakeholders in the Shelter and Settlements Sector. It also intends to follow the guidance for future standards set out by Hirano, Knudsen, and Serdaroglu (2018, p. 171) in *The State of Humanitarian Shelter and Settlements 2018,* which suggests that '[h]elpful humanitarian standards are developed through consensus, informed by the most current technical knowledge and practice, drawing upon global experience, and refined to be locally applicable'.

## The importance of defining shelter

Definitions are used to state the reference of a term. This has been explored in Philosophy through the logic of extensions and intensions, whereby the intension is the list of attributes an object must have to be labelled with the term, and the extension is the collection of objects to which the term applies. The intensional meaning of a word can thus determine its extension.

In addition to extension and intension, the meaning of any term may be understood through connotation and denotation. Connotation is the array of positive and negative associations connected with or implied by a term, and denotation is the precise, literal application of a term. As a result, there are two methods to approach the definition of a general term: as a denotative definition; or as a connotative definition. A denotative definition identifies the complete extension of the term in question. However, Lam (2016, p. 21) points out that ‘a complete enumeration of the things to which a general term applies would be cumbersome or inconvenient in many cases’. The alternative, therefore, is a connotative definition, which ‘tries to identify the intension of a term by providing a synonymous linguistic expression or an operational procedure for determining the applicability of the term’, offering ‘an adequate means for securing the meaning of a term’ (Lam, 2016, p.21). This paper will focus on developing a connotative definition, which outlines the associations implied by ‘shelter’. This definition will seek to determine the attributes ‘shelter’ must have and consequently, inform the extension of the term within the Sector.

It is important to develop a new definition because current terminology has led to much criticism and there is evidence it is hindering progress. Boano and Hunter (2012, p. 3) refer to shelter terminology as ‘profoundly semantic confusion’ that requires ‘deciphering nuances… as the consequences of conceptual confusion may create unwelcome results’. This is reflected in Zyck and Kent (2014) who surveyed 203 stakeholders across the private sector and within humanitarian aid on the role of business in ‘[h]umanitarian crises, emergency preparedness and response’. In this research, ‘two‐thirds of the aid workers and business figures surveyed in the project felt strongly that collaboration between relief agencies and business was often undermined by conflicting vocabularies and unclear terminologies’ (Zyck and Kent, 2014, p. 7). What is more, in a recent review of shelter terminology and typologies, Brogden and Kennedy (2021, p. 3) assert that ‘the proliferation of shelter terminology and its inconsistent use in the shelter sector impedes development and obstructs new actors. Terminology influences the implementation of coherent sector principles and inconsistent use is a barrier to meaningful engagement from new partners seeking to access shelter‐sector knowledge. Further, misunderstood terminology limits the development of new strategic approaches and innovation’. It is clear, therefore, that confusion of the terminology surrounding shelter has had, and will probably continue to have, detrimental effects.

Dean et al. (2016, p. 55) state that ‘the presentation of an alternative definition may appear generic, even unimportant’, but it can be Very important' because of ‘the potential to provide clarity’. They write that ‘at their best, definitions act like a compass, providing a lost reader with several potential directions from which to proceed’ (Dean et al., 2016, p. 55). It could be argued that the current compass directing the Shelter and Settlements Sector needs improvement. A new definition, incorporating progressions in the Sector, can deliver clarity to both internal and external actors and new, adequate means for comprehending how shelter is defined in the present day. Furthermore, within the humanitarian aid system, a singular definition can provide an immediate guide to stakeholders influencing decision‐making, such as governments, donors, and policymakers who may not be continuously involved in or aware of the changes and progressions specific to the Sector. A singular definition could help to move initial assumptions away from prior notions of shelter as a product towards understanding the different role of sheltering as a process.

## A history of shelter definitions in humanitarian aid

Within the prominent literature surrounding humanitarian aid, the definition of a shelter first appears in the *Multilingual Glossary of Human Settlement Terms:*

*Shelter, adequate: Immediate environment for all aspects of family life, providing protection from the elements, secure tenure, personal safety, access to clean water and sanitation, proximity to places of employment and educational and health care facilities* (United Nations Centre for Human Settlements (Habitat), 1992, p. 121).

Sometimes used interchangeably with shelter, the definition of housing, within the same document, stretches only to cover the immediate physical environment:

*Housing: Lodging, shelter for human habitation. The immediate physical environment, both within and outside of buildings, in which families and households live and which serves as shelter* (United Nations Centre for Human Settlements (Habitat), 1992, p. 61).

Conversely, Quarantelli (1995) is the first to make a distinction between shelter and housing, whereby *sheltering* refers to a place to stay in the immediate aftermath of disaster, adjourning daily activities, and *housing* indicates a return of household responsibilities and routine (see also Johnson, 2007). This would appear to be an inversion of the *Multilingual Glossary of Human Settlement Terms,* demonstrating the confusion within the terminology of the Sector. Quarantelli (1995) also goes further, reflecting on an earlier study, and defining the four key stages of sheltering as ‘emergency sheltering’, ‘temporary sheltering’, ‘temporary housing’, and ‘permanent housing’. However, these stages are later criticised for dissecting the process of sheltering into stages rather than remaining a continuum, and for failing to account for some typologies (Peacock, Dash, and Zhang, 2007).

In 1996, the definition of ‘adequate shelter’ was brought into international law in the development sector by the United Nations (UN) through the Habitat Agenda and the Istanbul Declaration on Human Settlements. Although it is not legally binding, this definition, different to that in the UN's 1992 glossary, provided clarity that *‘[a]dequate shelter means more than a roof over one's head‘* (United Nations Secretariat, 1996, p. 26). It lays out a series of provisions which make up the basis of adequate shelter:

*[Adequate shelter] means adequate privacy; adequate space; physical accessibility; adequate security, including security of tenure; structural stability and durability; adequate lighting, heating and ventilation; adequate basic infrastructure, such as water‐supply, sanitation and waste‐management facilities; suitable environmental quality and health‐related factors; and adequate and accessible location with regard to work and basic facilities: all of which should be available at an affordable cost* (United Nations Secretariat, 1996, p. 26).

Hurkmans (2018) commented on the variety of definitions of adequacy since this statement in *The State of Humanitarian Shelter and Settlements 2018*. He asserts that ‘different adequacy indicators may sometimes conflict with each other’ and that ‘[m]ost shelter actors agree that a one‐size‐fits‐all definition of adequacy is almost impossible’ (Hurkmans, 2018, p. 174). Since the publication of the UN‐Habitat (United Nations Human Settlements Programme) definition, Hurkmans (2018) notes that the Sphere Project, the United Nations Refugee Agency (UNHCR), and the Global Shelter Cluster have all modified it to some degree. However, it was still suggested that the elements outlined in the 1996 definition were most applicable to different contexts (Hurkmans, 2018).

In the late 1990s and early 2000s, there was growing recognition of the lack of research undertaken in shelter and housing, with Quarantelli's shelter and housing typology, published in 1982 and reviewed in 1995, forming the basis for most academic research. In 2007, Peacock, Dash, and Zhang pointed out that in the 20 years following Quarantelli's typology, which offered seven high‐priority topics for research, ‘two have received no attention, four have received limited attention, and only one has received, relatively speaking, a good deal of attention albeit by only a few researchers’ (Peacock, Dash, and Zhang, 2007, p. 260).

In 2005, however, Corsellis and Vitale published *Transitional Settlement, Displaced Populations,* intended to ‘help co‐ordinators and specialists working in humanitarian relief… [with] developing and implementing settlement and shelter strategies for people affected by conflict or natural disaster’ (Corsellis and Vitale, 2005, p.1). This also included a way ‘to help specialists to communicate with each other and understand one another’ (Corsellis and Vitale, 2005, p.6). Corsellis and Vitale (2005) argue that, rather than this varying terminology of shelter, there should be a movement towards the term *transitional settlement* as an alternative to the *shelter sector*. This would broaden the scope to include ‘settlement and shelter resulting from conflict and natural disasters, from emergency response to durable solutions’ (Corsellis and Vitale, 2005, p. 10). The benefit of this terminology, according to Corsellis and Vitale (2005), is to encompass both traditionally defined refugees and the response to similar needs of non‐refugees and internally displaced persons (IDPs), as well as acknowledging operational activities rather than just the physical structure itself. Within this text they defined shelter as:

*A habitable covered living space, providing a secure, healthy living environment with privacy and dignity to those within it* (Corsellis and Vitalle, 2005, p.11).

This statement has since been adapted in various forms and echoed across multiple handbooks, academic papers, funding proposals, guidance notes, articles, and manuals in the Sector. Some of the uses and variations of this definition over the past 10 years are summarised in Table 1. The Global Protection Cluster Working Group (2010, p. 236) also states that, '[w]hen humanitarian organizations refer to shelter, they generally mean habitable, covered living space, providing a secure and healthy living environment with privacy and dignity'. This definition, and variations on it, therefore, have become one of the most commonly used ways to define shelter to date.

**Table 1:** Adaptations of Corsellis and Vitalle's (2005) definition of shelter

Organisation	Year	Document	Definition*
International Federation of Red Cross and Red Crescent Societies	2009	*Shelter Kit Guidelines*	‘Definition of shelter. Shelter is more than a roof! A shelter is a secure **habitable covered living space** providing **privacy** and **dignity** for those within it’ (IFRC, 2009, p. x).
Global Protection Cluster Working Group	2010	*Handbook for the Protection of Internally Displaced Persons*	‘When humanitarian organizations refer to shelter, they generally mean **habitable, covered living space,** providing a **secure** and **healthy living environment** with **privacy** and **dignity‘** (Global Protection Cluster Working Group, 2010, p. 236).
Shelter Centre	2010	*Literature Review for Shelter After Disaster*	‘The basic definition of shelter is a **habitable covered space** providing a **secure** and **healthy environment** with **privacy** and **dignity** for those residing in the dwelling’ (Shelter Centre with Royal Roads University and FP Innovations, 2012, p.vii).
Department for International Development, United Nations Office for the Coordination of Humanitarian Affairs, and Shelter Centre	2010	*Shelter After Disaster*	‘[Transitional shelter] provides **a habitable covered living space** and a **secure, healthy living environment,** with **privacy** and **dignity,** for those within it, during the period between a conflict or natural disaster and the achievement of a durable shelter solution’ (DFID, UN OCHA, and Shelter Centre, 2010, p. 323).
Danish Refugee Council	2012	Project document; funding request; MPTF (Multi‐Partner Trust Fund) Office	‘The Somalia Shelter Cluster advocates for a transitional approach for shelter provision, offering a **habitable covered living space** and a **secure, healthy living environment,** with **privacy** and **dignity‘** (Danish Refugee Council, 2012, p. 1).
Shelter Recovery and Reconstruction Working Group	2013	*SSRR Definitions, Version 3*	‘Shelter is the process of providing a **“habitable covered living space,** providing a **secure, healthy living environment** with **privacy** and **dignity** to those within it”’ (Shelter Recovery and Reconstruction Working Group, 2013, p. 1).
UNHCR (United Nations Refugee Agency)	2014	*Global Strategy for Settlement and Shelter: A UNHCR Strategy 2014–2018*	‘[A shelter is] a **habitable covered living space** providing a **secure** and **healthy living environment** with **privacy** and **dignity‘** (UNHCR, 2014, p. 22).
SciDev.Net	2015	*Shelter after Disaster: Facts and Figures*	‘Shelter: A **habitable, covered living space** that provides a **secure** and **healthy living environment,** with **privacy** and **dignity** for people who reside within it’ (Wolfe Murray, 2015).
UNHCR	2015	*Emergency Handbook*	‘A shelter is defined as a **habitable covered living space** providing a **secure** and **healthy living environment** with **privacy** and **dignity‘** (UNHCR, 2015, p. 1).
UNHCR	2016	*Shelter Design Catalogue*	‘A shelter is defined as a **habitable covered living space** providing a **secure** and **healthy living environment** with **privacy** and **dignity‘** (UNHCR, 2016, p. 5).
DG ECHO (Directorate‐General for European Civil Protection and Humanitarian Aid Operations)	2017	*Humanitarian Shelter and Settlement Guidelines*	‘Shelter: A **habitable covered living space** providing a **secure, healthy living environment** with **privacy** and **dignity** to the groups, families, and individuals residing with[in] it’ (DG ECHO, 2017, p. 98).
Sphere Association	2018	*The Sphere Handbook*	‘Shelters and settlements are inter‐related and need to be considered as a whole. “Shelter” is the **household living space,** including the items necessary to support daily activities. “Settlement” is the wider locations where people and community live…. In addition to providing protection from weather, shelter is necessary to **promote health,** support family and community life, and **provide dignity, security** and access to livelihoods’ (Sphere Association, 2018, p. 240).
Global Shelter Cluster	2018	*Shelter and Settlement: The Foundation of Humanitarian Response. Strategy 2018–2022*	‘The primary objective of shelter response is safeguarding the **health, security, privacy** and **dignity** of affected populations. Shelter is a **physical component** of protection. Beyond life‐saving objectives, shelter also increases resilience, supports family and community life and facilitates access to livelihoods and markets’ (Global Shelter Cluster, 2018, p. 12).
Office of Disaster Management in the Commonwealth of Dominica, IOM (International Organization for Migration), and USAID (United States Agency for International Development)	2019	*Emergency Shelter Management Manual: For Shelter Managers and Coordinators in the Commonwealth of Dominica*	‘In any case, emergency shelters: need to be ready for disaster; need to be **habitable,** with adequate **covered living space;** need to provide a **secure** and **healthy living environment** with **privacy** and **dignity‘** (Office of Disaster Management, 2019, p. 16).
InterAction and USAID	2020	*Humanitarian Shelter and Settlements Training Course*	‘Shelter provides safety, **security, health, dignity,** and **wellbeing,** and thus can generate the impacts needed for response and recovery’ (InterAction and USAID, 2020).
Global Shelter Cluster and DMS (Displacement Management System)/CCCM (Camp Coordination and Camp Management) Nigeria	2021	*Guidance Note on Transitional Shelter*	‘Transitional shelter solution. Definition: provides a **habitable covered living space** and a **secure, healthy living environment,** with **privacy** and **dignity,** to those within it, during the period between a conflict or natural disaster and the achievement of a durable shelter solution’ (Global Shelter Cluster and DMS/CCCM Nigeria, 2021, p. 1).

**Note:** * Emphasis added.

**Source:** authors.

However, since its publication, the Sector has not been without other definitions and there has been a continual debate about distinguishing between shelter and housing. In 2007, in the *Handbook of Disaster Research,* Peacock, Dash, and Zhang (2007) undertook an extensive review of research in the sector. They recognised first that shelter is commonly defined as a temporary measure, usually expected to be ‘short term’. However, they also acknowledged that ‘no one has defined exactly what short term entails’ (Peacock, Dash, and Zhang, 2007, p. 262). In addition, they reasserted the distinction between sheltering and housing as the resumption of household activities and responsibility, suggesting that ‘with temporary housing, routine day‐to‐day household activities are re‐established’ (Peacock, Dash, and Zhang, 2007, p. 263).

The initial point about timing is echoed in some other later definitions of shelter in the Sector. In 2013, Sanderson and Burnell (2013, p.189), in answer to the question 'what is “shelter after disaster”?' in *Beyond Shelter after Disaster,* stated that 'the term refers to temporary structures beyond tents, and efforts by aid agencies to construct permanent post‐disaster housing'. This statement has a focus on both the ‘temporary’ nature and on ‘structures’ or ‘construction’. In 2020, InterAction and the United States Agency for International Development (USAID) released an online training module as part of *Shelter and Settlements* in which they point out that ‘while housing is the term used in the development sector, humanitarians use the word shelter, partly to signify its non‐permanent nature and to clarify the intention to support provision of dwellings that have limited lifespan’ (InterAction and USAID, 2020). However, there are also suggestions that shelter can have a longer‐term lifespan. Davis and Parrack (2018, p. 9) suggest that a ‘better understanding of shelter and settlements’ can be gained through ‘consider[ing] changes over time, and to recognize the scope of the subject’. They argue that a longer‐term perspective can help to address challenges.

The argument of ‘housing’ versus ‘shelter’ was further addressed by Ian Davis and David Alexander in 2016, as ‘house’ versus ‘home’. They make reference to Paul Oliver's work on the anthropology of shelter, in which he made the following distinction:

*A town is made of buildings, but a community is made of people; a house is a structure, but a home is much more. The distinctions are not trivial, nor are they sentimental or romantic: they are fundamental to the understanding of the difference between the provision of shelter which serves to protect and the creation of domestic environments that express the deep structures of society* (Oliver, 2006, in Davis and Alexander, 2016, p. 187).

Oliver's distinction could be interpreted as suggesting that the recognition of shelter as merely a structure fundamentally undermines the creation of spaces that the earlier UN‐Habitat definition would describe as ‘adequate’. Indeed, as previously stated *‘adequate shelter means more than a roof over one's head‘* (United Nations Secretariat, 1996, p. 26). Davis and Alexander (2016, p. 188) make a further point about the personalisation of temporary shelter and draw attention to the fact that even where ‘little scope existed for personalising… it was nevertheless done’. In 2018, the Sphere Association (2018, p. 240) echoed the need to define shelter and settlement response beyond just providing structures, stating that:

*Shelter and settlement response options are not limited to delivering hardware and materials or constructing a shelter. Response options also include providing support to secure land and obtain shelter, housing or household items. This includes technical assistance and quality assurance, which can empower and mobilise an affected population to build back better and more safely*.

This Sphere Standard drew input from a wide group of practitioners, cocreated by actors from the Shelter Sector, and thus reflecting the state of shelter and settlements at the time.

According to the Sphere Standard's definition of shelter, listed within ‘Essential concepts in shelter and settlement’, the approach adopted is to separate shelter and settlements, with shelter being ‘the household living space, including the items necessary to support daily activities’, and settlement being ‘the wider locations where people and community live’ (Sphere Association, 2018, p. 240). This section then elaborates on the aim of shelter and settlement responses ‘to provide a safe living environment’ and that ‘in addition to providing protection from weather, shelter is necessary to promote health, support family and community life, and provide dignity, security and access to livelihoods’ (Sphere Association, 2018, p. 240). This definition contains elements of the earlier definition of Corsellis and Vitalle (2005), a household ‘living space’ with ‘health’, ‘dignity’, and ‘security’, while also expanding slightly further on the impacts of shelter with respect to incorporating support of family life and access to livelihoods. It is clear, when reading the shelter and settlements chapter that the process approach is advocated, in line with the progression of the Sector. However, to understand this approach, the first definition must be read in conjunction with more of the guidance.

The InterAction and USAID (2020) training module also recognised the wider provisions of shelter, again separating shelter and settlements:

*Shelter provides safety, security, health, dignity, and wellbeing, and thus can generate the impacts needed for response and recovery. It also contributes towards longer term disaster risk reduction, and therefore the resilience of a community. Settlements are the contexts for these activities. To understand the context is to be able to design an intervention that is effective, culturally appropriate, and creates long‐lasting impacts for affected communities*.

In 2021, Brogden and Kennedy (2021, p. 19) completed an analysis of humanitarian shelter terminology, producing a tool for ‘conceptualizing interconnected shelter phases, products, and processes’. This research condensed 347 terms for shelter into eight categories: ‘Immediate Shelter; Intermediate Shelter; Permanent Shelter; Pre‐Emptive Shelter; Non‐Specific Shelter Terms; Shelter Items; Alternative Strategies; Multiphase Shelter’ (Brogden and Kennedy, 2021, p. 13). These can be broken down into a further 25 subcategories, demonstrating the wide variety of possible interpretations of ‘shelter’ across the Sector, both material and nonmaterial.

However, Fekete et al. (2021, p. 4) also argue that humanitarian standards for shelter may not actually incorporate enough, suggesting ‘the aspect of potential failure of infrastructure is lacking [within the shelter topic]‘. They state:

*Shortcomings of purely structural and technical shelter planning are known for overseeing perspectives of social, psychological, ethnic and many other aspects. However, planning should still be advanced on awareness of dependency on infrastructure supply in a technical sense and aspects such as access of people, integration of civil society, infrastructure operators, local authorities, private contractors and many more stakeholders* (Fekete et al., 2021, p. 11).

This suggests that broader consideration of the local environment, stakeholders, communities, and infrastructure is also necessary.

It is clear from this variety of definitions and specification of provisions that shelter, and its wider role, can be perceived differently by different actors. It is crucial that these definitions receive clarity, continuity, and a comprehensive definition. This is useful not only for stakeholders such as agencies, donors, and academics, but also the communities that must live with the outcome of any perceived or determined notion of what shelter is and what it can do.

## Deconstructing the ‘habitable covered living space’

The Corsellis and Vitalle (2005) definition has been chosen for deconstruction owing to its proliferation across the Shelter and Settlements Sector. Table 1 illustrates that elements of this definition have been re‐used, adapted, and interpreted across literature in the Sector, including practitioner manuals, academic journals, training programmes, and funding proposals.

Before deconstructing the definition set out originally by Corsellis and Vitale (2005), one must first acknowledge that this definition has been very useful to the Sector and effective in ensuring better provision of shelter to affected communities. This deconstruction is not intended to take away from the success of this definition, but merely to highlight why it is necessary to update the definition to reflect the positive progression of the Sector. It is accepted that Corsellis and Vitalle's (2005) definition of shelter came a long distance in moving the Sector away from the staged approach initially suggested by Quarantelli (1995) and the confusion between ‘shelter’ and ‘housing’ to a more concise and precise definition of shelter.

Corsellis and Vitalle's (2005, p. 9) definition is as follows:

*A habitable covered living space, providing a secure, healthy living environment with privacy and dignity to those within it*.

There are several reasons why this definition is now somewhat outdated. First, it lacks clarity on Davis' (1978) concept of ‘process’ rather than ‘object’. The reference to ‘a habitable covered living space’, for instance, can be directly interpreted as an object. While it is clear that within the context of *transitional settlements,* Corsellis and Vitalle (2005) are proponents of the ‘process’ approach, out of context, the definition can be construed as relating solely to an object. It requires the knowledge of the reader to assume or imply that shelter can be process‐driven, and not just seen through the lens of an object. As such, the intension of the definition does not necessarily lead to its extension.

Second, there is some ambiguity regarding the provisions provided by the ‘habitable covered living space’. By dictating the four provisions of security, health, privacy, and dignity (which are then commonly repeated in other interpretations), the definition relies too heavily on the reader for two purposes. First, the reader must assign their own definition to each of these provisions. However, several terms included in this definition have been debated. The concept of ‘dignity’ is included in many references within humanitarian aid, but Holloway and Grandi (2018, p. 21) highlight that although it is frequently used by humanitarian actors, they ‘rarely define its meaning explicitly’ and there is a need ‘to further explore the concept of dignity and what it means in practice in particular locations and crises’. In addition, some shelter situations have been shown to affect negatively the concepts of ‘dignity’ and ‘privacy’ referred to in the definition. In a review of shelter conditions, the IRC (2017, p. 2) stated that ‘cramped communal shelter conditions are perceived by communities to increase the risk of intimate partner violence and child marriage, while particularly affecting privacy, dignity and psychosocial well‐being for women’ (see also Holloway and Grandi, 2018). Furthermore, it could also be perceived that it is without the capacity of humanitarian aid actors to confer dignity on to any person or community, and likewise, living conditions may not form the basis for whether an individual considers that they possess dignity. This stance is arguably also asserted in Article 1 of the *Universal Declaration of Human Rights:* ‘All human beings are born free and equal in dignity and rights’ (UNGA, 1948). Privacy is also contentious and ambiguous in the definition. For instance, what may be considered as ‘privacy’ in the traditionally shared homes of generations of Hindu Indonesian families as compared to those of more conservative Muslim Indonesian families can be vastly different, and again therefore, self‐defined rather than conferred.

Second, the reader must implicitly know to search for provisions outside of these four provisions when compared to the UN‐Habitat definition of adequate shelter. A simple example of this could be economic provisions such as affordability or livelihoods. While this is not a problem for individuals who are well versed in Sector‐related needs, it presents barriers to the successful provision of shelter when decision‐makers are new and/or rely solely on addressing these four aspects outlined in the definition. More recent adaptations of the definition seem to recognise this issue and include additional considerations, such as in the *Sphere Handbook* and Global Shelter Cluster Strategy 2018–2022. However, this non‐collective, organic development has led to a lack of consistency, with organisations still catching up and some continuing to use the original definition, as seen in Table 1. Many of these definitions can also be interpreted as denotative definitions, attempting to list the complete extension of the term ‘shelter’, and failing to do so.

Lastly, the definition lacks clarity on for whom these provisions are intended and their potential wider effects. ‘To those within it’ can encompass individuals, families, and communities, but this grouping fails to acknowledge the wider host communities and the environment in which the shelter is placed. When discussing ‘shelter in displacement’, Chief of Shelter and Settlement at UNHCR and Global Shelter Cluster co‐lead, Brett Moore (2017, p. 5) suggests that the ‘attitude of host communities, security concerns and the willingness of a host government to meet their obligations have a direct impact on the viability and adequacy of refugee settlements’. Furthermore, he notes that ‘the aspirations of the refugee community and the host community need to be jointly taken into consideration for any long‐term solution’ (Moore, 2017, p. 7). The need to pay attention to host communities and recognise the role of the host state is also echoed throughout guidance such as the *Sphere Handbook* (Sphere Association, 2018).

## Constructing a new definition

Following on from the range of definitions in the Sector, we decided to focus on the definition of shelter rather than housing. Although it is clear that housing has been utilised in the past, the distinction is made in this instance along the lines of the humanitarian and the non‐humanitarian context. This is to say that shelter refers to the response to a crisis (that is, a disaster, conflict, or complex emergency) whereas housing is the form of shelter outside of this setting.

In reviewing the previous definitions and Sector progressions, five key aspects were identified in the literature as important to incorporate in the new definition. These are as follows:

1. To reflect the concept of **process** over object (Davis, 1978).
  This discussion has become a central tenet of the Shelter and Settlements Sector. As it stems from ‘housing as a verb’ (Turner, 1972), the new, proposed definition of shelter is addressed through ‘sheltering as a verb’. To avoid ambiguity, the concept of process is also explicitly referred to and the role of the Sector within that process has been further considered. As suggested by multiple sources, it should be one of enabling communities (Karunasena and Rameezdeen, 2010; Maly, 2018; Babister, 2020). This can yield various outputs, including technical guidance and capacity‐building.
2. To reflect that the needs of shelter affect **communities** as well as individuals (Corsellis and Vitale, 2005).
3. To reflect the often longer‐term nature of sheltering and related needs (Davis and Parrack, 2018).
4. To reflect that shelter is often at **the centre of wider needs** (Kelling, 2020).
  The needs of individuals may differ from those of communities, as well as between contexts and along timelines. To ensure that the new definition encompasses those wider needs and takes a ‘long‐term view’, the definition has been structured in a way that recognises the potential for change, and the possibility of extending beyond the temporary. It does not list the needs as previous definitions have done because of the risk of creating an inexhaustive list and because these should be defined by members of the affected population themselves.
5. To reflect that shelter also affects the **host communities** and the wider **environment** (UN‐Habitat, IFRC, and UNHCR, 2008, 2012, 2013; Moore, 2017).
  Finally, the definition should recognise that shelter projects can have a bearing on the host communities and the local environment, as well as on the crisis‐affected populations that they serve to assist. This can be through, for example, the increased demands placed on critical infrastructure (Fekete et al., 2021). The environment can be understood as both the surroundings in which the sheltering may take place and the natural habitat within a particular geographical area.

The complete, proposed definition is as follows: *sheltering is an enabled process to facilitate a living environment with crisis‐affected communities and individuals, to meet their current and future needs, whilst also having due consideration for the needs of the host communities and environment*. Given the importance of the UN‐Habitat definition of ‘adequate shelter’ and recent agreement on its applicability in *The State of Humanitarian Shelter and Settlements 2018* (Sanderson and Sharma, 2018), the ‘needs’ in the definition above should be guided by those that are highlighted by UN‐Habitat in combination with those articulated by the crisis‐affected communities and agreed with the line ministries of host governments, with whom strategic guidance is often produced for sheltering activities.^1^ Furthermore, in accordance with the UN‐Habitat definition, ‘[a]dequacy should be determined together with the people concerned, bearing in mind the prospect for gradual development’ (United Nations Secretariat, 1996, p. 26). This definition is intended to be connotative, identifying the intension of the term ‘shelter’ in an adaptable format, while ensuring that it does not result in an inexhaustive checklist.

## Conclusion

Hirano, Knudsen and Serdaroglu (2018, p. 171) write in *The State of Humanitarian Shelter and Settlements 2018* that ‘[s]tandards are not something that can be kept on the shelf; nor are they an abstract concept. The weight of a standard lies in its power to translate fundamental rights and principles into actions that save lives, protect dignity and promote recovery’. It is clear, then, that the weight of this definition and the primary impact of changes to the fundamental way we view and define shelter will most affect the communities that must live through the process of sheltering, of redefining their home, and of re‐establishing their needs over time. It is also clear, therefore, that the needs of affected populations and standards of adequacy can only be defined by the populations themselves, aided by the technical guidance of expertise in the Sector. The purpose of this new definition is to reflect recognition of this within the Sector, and to place affected communities at the heart of any sheltering process. This definition should also be viewed as a reflection of the current environment and as such, it is recommended that it be kept under coordinated review, to respond to and evolve with future changes across the humanitarian landscape. One opportunity for this may be during the regular development and review of the Global Shelter Cluster's five‐year strategy.

The definition is likely to be of direct use and relevance to professionals engaged in the sheltering process, particularly governments, donors and policymakers determining high‐level strategies for shelter, but always with the needs and considerations of the affected people uppermost in mind. If the needs of the affected populations define the process of sheltering, it is clear that canvassing and understanding of those needs must be the first stage in any shelter and settlements response. It is only from that point that the Shelter and Settlements Sector may then assist in enabling recovery, as merely a deuteragonist in a long‐term process. The principal focus should always be recognised as, and remain throughout, the affected populations.

## Acknowledgements

The authors gratefully acknowledge the Royal Commission for the Exhibition of 1851 and the Worshipful Company of Constructors for funding this research, alongside the additional support from the Engineering and Physical Sciences Research Council's Centre for Doctoral Training in Future Infrastructure and Built Environment (grant reference number: EP/L016095/1).

## Data availability statement

Data sharing is not applicable to this article as no new data were created or analyzed in this study.

## Endnotes

1 This is in line with United Nations General Assembly Resolution 46/182 (UNGA, 1991), which underpins host government involvement in defining the response and setting out the frameworks to govern shelter assistance, with the aid of the Inter‐Agency Standing Committee.
